# 3,3′-Diindolylmethane Suppressed Cyprodinil-Induced Epithelial-Mesenchymal Transition and Metastatic-Related Behaviors of Human Endometrial Ishikawa Cells via an Estrogen Receptor-Dependent Pathway

**DOI:** 10.3390/ijms19010189

**Published:** 2018-01-08

**Authors:** Bo-Gyoung Kim, Jin-Wook Kim, Soo-Min Kim, Ryeo-Eun Go, Kyung-A Hwang, Kyung-Chul Choi

**Affiliations:** Laboratory of Biochemistry and Immunology, College of Veterinary Medicine, Chungbuk National University, Cheongju 28644, Chungbuk, Korea; snubbomed@naver.com (B.-G.K.); kimjinewook@gmail.com (J.-W.K.); tnals1613@gmail.com (S.-M.K.); gmyich@naver.com (R.-E.G.)

**Keywords:** cyprodinil (CYP), 3,3′-diindolylmethane (DIM), epithelial-mesenchymal transition (EMT), metastasis

## Abstract

Cyprodinil (CYP) is a pyrimidine amine fungicide that has been extensively used in agricultural areas. 3,3′-Diindolylmethane (DIM) is a derivative of the dietary phytoestrogen, indole-3-carbinol (I3C), which is derived from cruciferous vegetables and considered to be a cancer-preventive phytonutrient agent. In this study, the effects of CYP and DIM were examined on the cell viability, invasion, and metastasis of human endometrial cancer cells, Ishikawa, via epithelial mesenchymal transition (EMT). CYP increased the level of cell viability of Ishikawa cells compared to DMSO as a control, as did E2. Ishikawa cells lost cell-to-cell contact and obtained a spindle-shaped or fibroblast-like morphology in response to the application of E2 or CYP by the cell morphology assay. In the cell migration and invasion assay, CYP enhanced the ability of migration and invasion of Ishikawa cells, as did E2. E2 and CYP increased the expressions of N-cadherin and Snail proteins, while decreasing the expression of E-cadherin protein as EMT-related markers. In addition, E2 and CYP increased the protein expressions of cathepsin D and MMP-9, metastasis-related markers. Conversely, CYP-induced EMT, cell migration, and invasion were reversed by fulvestrant (ICI 182,780) as an estrogen receptor (ER) antagonist, indicating that CYP exerts estrogenic activity by mediating these processes via an ER-dependent pathway. Similar to ICI 182,780, DIM significantly suppressed E2 and CYP-induced proliferation, EMT, migration, and invasion of Ishikawa cancer cells. Overall, the present study revealed that DIM has an antiestrogenic chemopreventive effect to withdraw the cancer-enhancing effect of E2 and CYP, while CYP has the capacity to enhance the metastatic potential of estrogen-responsive endometrial cancer.

## 1. Introduction

Phytoestrogens of plant origin, such as plant polyphenols, are xenoestrogens that show structural similarity to 17β-estradiol (E2), the mammalian steroid hormone [1]. Phytoestrogens are generally categorized according to four main classes. The first group is isoflavones, such as daidzein, kaempherol, and genistein, while the second group consists of lignans, such as lariciresinol, matairesinol, pinoresinol, and secoisolariciresinol. The third group consists of coumestans, such as coumestrol, and the last comprises stilbenes, such as resveratrol [2]. Among plant-derived xenoestrogens, phytoestrogens are primarily found in fruits, soy, and vegetables. Phytoestrogens are also regarded as sources of cancer-preventive phytonutrient complex because they inhibit the growth and advance of many types of cancer [3,4,5]. For example, genistein, a major soy isoflavone, and 3,3′-diindolylmethane (DIM), a derivative of the dietary phytochemical complex, indole-3-carbinol (I3C), which is derived from cruciferous vegetables, are phytoestrogens known for reducing the risk of prostate and breast cancer [6,7]. 3,3′-diindolylmethane has been reported to influence the prevention of estrogen-dependent cancers similar to fulvestrant (ICI 182,780), an estrogen receptor (ER) antagonist [8]. Moreover, the in vitro effects of DIM were shown to inhibit epithelial-mesenchymal transition (EMT) and metastasis via the estrogen receptor (ER)-dependent pathway [9]. Previous studies have shown that anti-estrogenic effects of phytoestrogens are implicated in their chemoprevention activity against estrogen-dependent cancers via the ER-dependent pathway [10,11,12].

The EMT is an adjusted process that drives epithelial cells to lose their cell-cell and cell-extracellular matrix (ECM) interactions and to become mesenchymal cells through genetic reprogramming and cytoskeletal restructuration [13]. The EMT has potential driving forces in the initiation and development of cancer cells [14]. Moreover, the EMT phenotype has advanced migratory capacity, invasiveness, and increasing resistance to apoptosis [15]. Cancer cells that undergo EMT augment the extent of expression of cell motility-related proteins and present improved invasion and migration to other parts of the whole body, resulting in cancer metastasis [16].

It is generally agreed that estrogen plays a significant role in cancer metastasis. For example, estrogens and endocrine disrupting chemicals (EDCs) such as benzophenon-1 and nonylphenol spur metastasis through overexpression of cathepsin D in MCF-7 breast cancer cells via the ER-dependent signaling pathway [17,18]. As a lysosomal aspartyl protease, cathepsin D is related to the metastasis of estrogen-dependent cancer cells [19]. In other examples, BP-1 and octylphenol have been found to induce EMT of BG-1 ovarian cancer cells expressing ERs [20]. In addition, bisphenol compounds can give rise to EMT of BG1Luc4E2 ovarian cancer cells expressing ERs [21].

Cyprodinil (CYP) is an extensive pyrimidine amine fungicide that is utilized worldwide to protect fruit plants and vegetables from many types of pathogens [22]. In fungi, this reagent prevents the biosynthesis of methionine and amino acids of thionic types [23]. CYP gives rise to phosphorylation of the extracellular signal-regulated kinase (ERK) by which growth factors and transcription factors are phosphorylated. In mammalian cells, ERK regulates differentiation, migration, proliferation, and survival [24], and activates ER signaling [25]. A previous study found that CYP as an activator of aryl hydrocarbon receptor (AhR) and induces AhR-targeted genes, such as *cytochrome P450* (*CYP*) *1A1* in ovarian granulosa cells, *HO23*, and potentially affects reproductive function through activating both the AhR and ERK signaling [26]. Additionally, CYP was found to have the potential to affect ER signaling in our previous study in which it promoted ovarian cancer proliferation via the ER-dependent pathway [24]. Therefore, it can be estimated that CYP may act as a cellular physiological disrupter in the human body.

The present study was conducted to investigate the CYP’s action as an endocrine disrupter by examining its xenoestrogenic effects on cancer cell proliferation, EMT, and metastasis by using an ER-dependent and estrogen-responsive Ishikawa endometrial cancer cell line, which is a well-differentiated adenocarcinoma cell line derived from the human endometrial epithelium that expresses functional ER [27]. In addition, anti-estrogenic and anti-cancer effects of DIM were investigated using this cancer model.

## 2. Results

### 2.1. Effects of CYP Exposure on Cell Viability of Ishikawa Endometrial Cancer Cells

**Figure 1:**
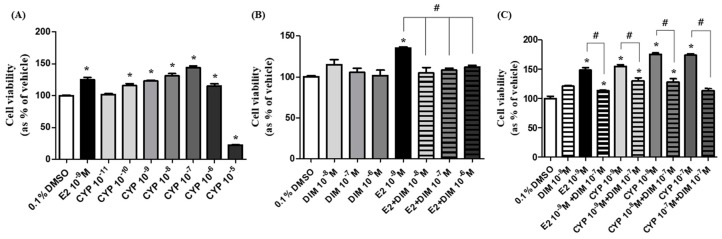
Effects of Cyprodinil (CYP) exposure on cell viability of Ishikawa endometrial cancer cells. Ishikawa cancer cells were treated with 0.1% DMSO as a control, E2 (10^−9^ M), CYP (10^−11^–10^−5^ M), or 3,3′-Diindolylmethane (DIM) (10^−8^–10^−6^ M) for six days, after which the cell viability was measured by MTT assay. The experiment was repeated three times, and data were reported as the means ± SD. (**A**) Effects of E2 and CYP on cell viability. * indicates a significant difference in cell viability by E2 or CYP compared to the control (*p* < 0.05 according to Dunnett’s multiple comparison test); (**B**) Effects of the mixture of E2 and DIM on cell viability. * shows a significant difference in cell viability by E2 or DIM compared to the control (*p* < 0.05 according to Dunnett’s multiple comparison test). # shows a significant reduction in cell viability in response to E2 + DIM compared to E2 alone (*p* < 0.05 according to Dunnett’s multiple comparison test); (**C**) Effects of the mixture of CYP and DIM. * shows a significant difference in cell viability in response to E2, DIM, CYP, E2 + DIM, or CYP + DIM compared to the control (*p* < 0.05 according to Dunnett’s multiple comparison test). # shows a significant reduction in cell viability in response to E2 + DIM compared to E2 alone or CYP + DIM compared to CYP alone (*p* < 0.05 according to Dunnett’s multiple comparison test).

This experiment was conducted to identify the effects of E2, CYP, and DIM on cell viability of Ishikawa cells and determine the optimal concentrations of E2, CYP, and DIM for subsequent experiments. As shown in Figure 1A, E2 (10^−9^ M) and CYP (10^−11^–10^−6^ M) augmented cell viability when compared with 0.1% DMSO as a control. Moreover, CYP increased cell viability in a concentration-dependent manner in the concentration range of 10^−10^–10^−6^ M, implying that CYP has an estrogenic effect at these concentrations. Although DIM did not change the cell viability at 10^−8^, 10^−7^, or 10^−6^ M, it inhibited E2- or CYP-induced cell viability when combined with E2 or CYP at these concentrations (Figure 1B,C). Based on these results, DIM was considered to have anti-estrogenic activity, contrary to CYP. Among the concentrations of CYP and DIM tested in this experiment, 10^−8^ and 10^−7^ M of CYP and DIM, respectively, were selected to evaluate the in vitro effects of each compound on the processes of EMT and metastasis of Ishikawa cells. Treatment with 10^−8^ M CYP increased the cell viability of Ishikawa cells to the same level as E2, a positive control (Figure 1A). DIM at 10^−7^ M was selected from 10^−8^, 10^−7^, and 10^−6^ M of DIM tested because there was no change in E2-induced cell viability at these concentrations (Figure 1C).

### 2.2. Morphological Changes in Ishikawa Cells in Response to Treatment with E2 and CYP in the Presence or Absence of ICI or DIM

**Figure 2:**
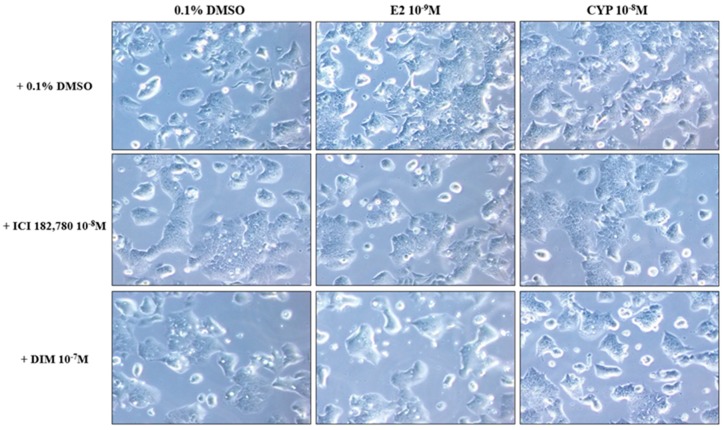
Morphological changes in Ishikawa cells in response to treatment with E2 and CYP in the presence or absence of ICI 182,780 or DIM. Ishikawa cells were cultivated in six-well plates and treated with E2 (10^−9^ M), CYP (10^−8^ M), DIM (10^−7^ M), or ICI 182,780 (10^−8^ M) for 24 h. Ishikawa cells were photographed using a microscope at a magnification of 400×.

To investigate the induction of EMT, morphological changes in Ishikawa cells in response to treatment with E2 (10^−9^ M) and CYP (10^−8^ M) in the presence or absence of DIM (10^−7^ M) or ICI 182,780 (10^−8^ M) were observed. After treatment for 24 h, microscopic analysis showed that Ishikawa cells lost cell-to-cell contact and developed a spindle- or a fibroblast-like morphology, which is a phenotype of mesenchymal cells, in response to treatment with E2 and CYP. Conversely, when treatment was applied in conjunction with ICI 182,780, or DIM, most Ishikawa cells maintained a cobblestone-like appearance, which is a typical morphology of epithelial cells (Figure 2). These results indicate that CYP mediated the induction of the EMT process of Ishikawa cells, similar to E2 via ER; however, DIM suppressed E2 or CYP-induced EMT process similar to ICI 182,780, an ER antagonist.

### 2.3. Effects of CYP and DIM on the Expression of EMT Related Genes

**Figure 3:**
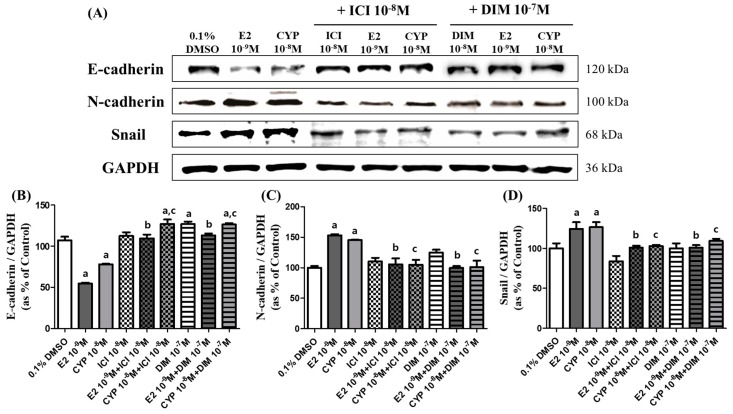
Effects of E2, CYP, ICI 182,780, and DIM on the expression of EMT related genes. Ishikawa cells were treated with 0.1% DMSO, E2 (10^−9^ M), CYP (10^−8^ M), a mixture of E2 (10^−9^ M) or CYP (10^−8^ M) and ICI (10^−8^ M), or a mixture of E2 (10^−9^ M) or CYP (10^−8^ M) and DIM (10^−7^ M), respectively, for 72 h. Total proteins were extracted and analyzed by Western blot. (**A**) Band images correspond to E-cadherin, N-cadherin, and Snail proteins. Quantification of bands corresponding to (**B**) E-cadherin, (**C**) N-cadherin, and (**D**) Snail proteins was conducted using Luminograph II. The experiment was repeated three times, and data are presented as the means ± SD. a: A significant augmentation or reduction in expression of each protein by E2 and CYP compared to the control (*p* < 0.05 according to Dunnett’s multiple comparison test); b: a significant reduction in the expression of each protein by the mixture with DIM or ICI and E2 compared to E2 alone (*p* < 0.05 according to Dunnett’s multiple comparison test); and c: a significant reduction in expression of each protein by the mixture with DIM or ICI and CYP compared to CYP alone (*p* < 0.05 according to Dunnett’s multiple comparison test).

The effects of each agent on the protein expressions of EMT-related genes including epithelial and mesenchymal cell markers were identified through Western blot assay. As shown in Figure 3, CYP (10^−8^ M) decreased the protein expression of E-cadherin, a key epithelial marker, by about 50%, which was similar to E2 (10^−9^ M), and by approximately 80% when compared to DMSO as a control (Figure 3A,B). Conversely, when ICI 182,780 (10^−8^ M) or DIM (10^−7^ M) was administered in conjunction with E2 (10^−9^ M) or CYP (10^−8^ M), the expression of E-cadherin was restored to the control level. Moreover, CYP (10^−8^ M) increased the protein expression of N-cadherin and Snail, which are mesenchymal markers, by about 45%, similar to E2 (10^−9^ M), which increased N-cadherin and Snail expression by 53% and 24%, respectively, compared to DMSO (Figure 3A,B). However, when applied in conjunction with ICI 182,780 (10^−8^ M) or DIM (10^−7^ M), the expression of N-cadherin and Snail returned to the control level. These results indicate that E2 and CYP induced the EMT process of Ishikawa cells by regulating the protein expression of EMT-related genes, such as E-cadherin, N-cadherin, and Snail, via the ER-dependent signaling pathway and that DIM inhibited the induction of the EMT process by neutralizing the effects of E2 and CYP on the protein expression of these genes.

### 2.4. Suppression of DIM on CYP-Induced Ishikawa Endometrial Cancer Cell Migration

**Figure 4:**
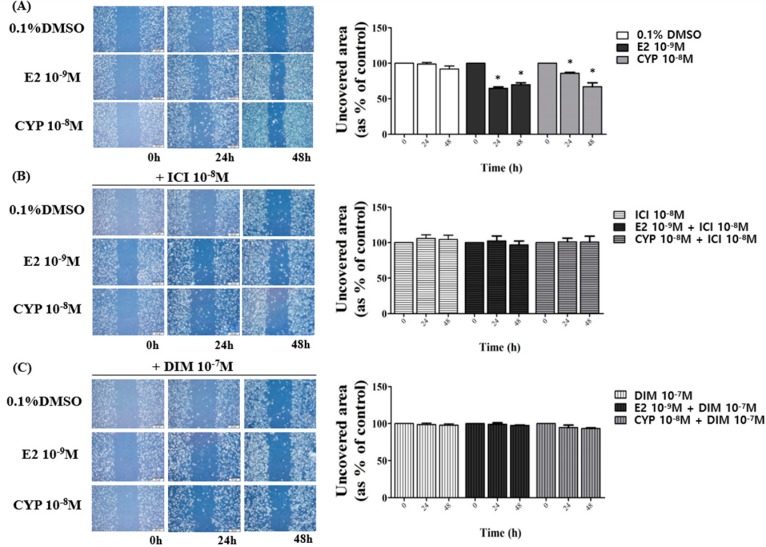
Effects of ICI 182,780 or DIM on E2- or CYP-induced Ishikawa endometrial cancer cell migration. Cells were treated with (**A**) 0.1% DMSO, E2 (10^−9^ M), CYP (10^−8^ M), (**B**) a mixture of E2 (10^−9^ M) or CYP (10^−8^ M) and ICI (10^−8^ M), or (**C**) a mixture of E2 (10^−9^ M) or CYP (10^−8^ M) and DIM (10^−7^ M), respectively, then scratched with a 1 mL micropipette tip. The images presenting the recovery of wounded area were captured at 0, 24, and 48 h using a microscope at a magnification of 40×. The percentage of the wound recovery area at each time point was calculated. The experiment was repeated three times, and data are presented as the means ± SD. *: Mean values were significantly differentiated from 0 h of each treatment, *p* < 0.05 (Dunnett’s multiple comparison test).

Changes in migration activity of Ishikawa cells treated with E2, CYP, DIM, and ICI 182,780 were identified by cell scratch assay. After the cells were treated with 0.1% DMSO as a control, E2 (10^−9^ M), CYP (10^−8^ M), DIM (10^−7^ M), or ICI 182,780 (10^−8^ M) and scratched using a 1 mL micropipette tip, wounded areas were photographed at 0, 24, and 48 h. Application of E2 or CYP reduced unrecovered wound areas in a time-dependent manner when compared to those of the control (Figure 4A), implying that CYP promoted the migration of Ishikawa cells as did E2.

After Ishikawa cells were treated with a combination of ICI 182,780 (10^−8^ M) or DIM (10^−7^ M) and E2 (10^−9^ M) or CYP (10^−8^ M), unrecovered areas were unchanged at 48 h (Figure 4B,C). According to these results, CYP may induce the migration of Ishikawa cells via the ER-dependent signaling pathway and DIM can inhibit the E2 or CYP-induced cell migration.

### 2.5. Suppression of DIM on CYP-Induced Ishikawa Endometrial Cancer Cell Invasion

**Figure 5:**
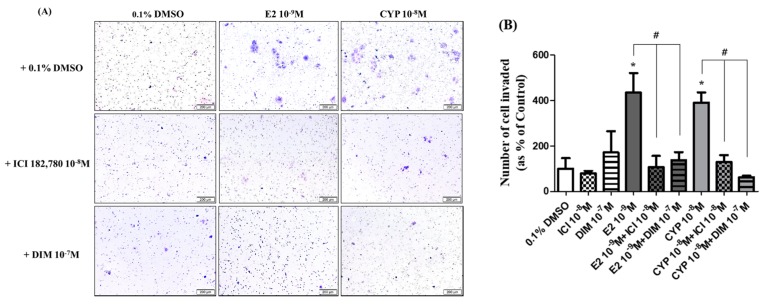
Effects of ICI 182,780 or DIM on E2- or CYP-induced Ishikawa endometrial cancer cell invasion. Ishikawa cells (1 × 10^5^ cells) were cultured in transwells with the bottom surface covered with fibronectin in each well of a 24-well plate for 24 h. Cells were treated with 0.1% DMSO, E2 (10^−9^ M), CYP (10^−8^ M), a mixture of E2 (10^−9^ M) or CYP (10^−8^ M) and ICI (10^−8^ M) or a mixture of E2 (10^−9^ M) or CYP (10^−8^ M) and DIM (10^−7^ M), respectively, for 24 h. The cells attached to the bottom surface of the transwell were fixed with 10% formalin solution and stained with crystal violet. (**A**) The stained cells were detected under a microscope, and (**B**) the number of invading cells was counted. The experiment was repeated three times, and data were presented as the means ± SD. *: Mean values were significantly different from 0.1% DMSO (control), *p* < 0.05 (Dunnett’s multiple comparison test). #: Mean values of the mixture of ICI 182,780 or DIM and E2 and the mixture of ICI 182,780 or DIM and CYP were significantly reduced from E2 or CYP alone (*p* < 0.05 according to Dunnett’s multiple comparison test).

To check the altered invasion capacity of Ishikawa cells in response to treatment with E2, CYP, DIM, and ICI 182,780, a transwell assay was conducted. After cells in the upper chamber of a transwell were treated with E2 (10^−9^ M) or CYP (10^−8^ M) for 24 h, the number of Ishikawa cells that moved from the top chamber to the bottom chamber was significantly augmented (Figure 5). Conversely, when treated with a mixture of ICI 182,780 (10^−8^ M) or DIM (10^−7^ M) and E2 (10^−9^ M) or CYP (10^−8^ M), the number of intruded cells was reduced to the control level (Figure 5A,B). These results indicate that CYP enhanced the invasion capacity of Ishikawa cells through an ER-dependent signaling pathway, as did E2, and that DIM has the capacity to restrain E2 or CYP-induced invasion of Ishikawa cells.

### 2.6. Effects of CYP and DIM on the Expression of Metastasis Related Genes

**Figure 6:**
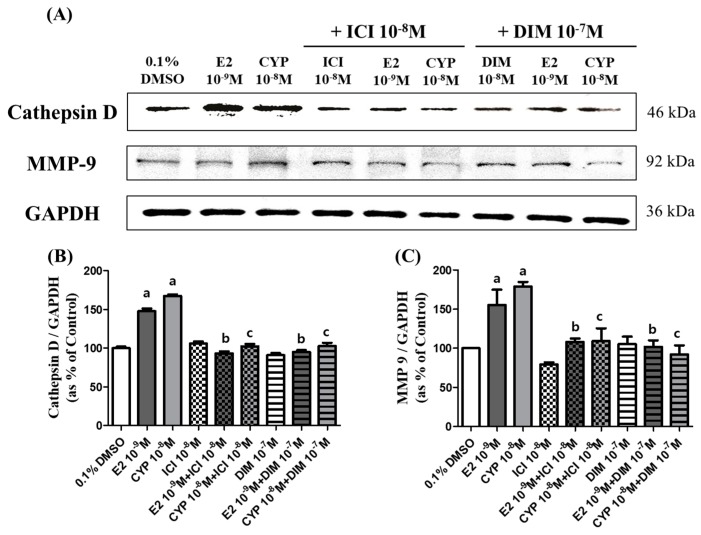
Effects of E2, CYP, ICI 182,780, and DIM on the expression of metastasis related genes. Ishikawa cells were treated with 0.1% DMSO, E2 (10^−9^ M), CYP (10^−8^ M), a mixture of E2 (10^−9^ M) or CYP (10^−8^ M) and ICI (10^−8^ M), or a mixture of E2 (10^−9^ M) or CYP (10^−8^ M) and DIM (10^−7^ M), respectively, for 72 h. Total proteins were extracted and analyzed by Western blot. (**A**) Band images correspond to Cathepsin D and MMP-9 proteins. Quantification of bands corresponding to (**B**) Cathepsin D and (**C**) MMP-9 proteins was conducted using Luminograph II. The experiment was repeated three times, and data are presented as the means ± SD. a: significant augmentation or reduction in expression of each protein by E2 and CYP compared to the control (*p* < 0.05 according to Dunnett’s multiple comparison test); b: significant reduction in expression of each protein by the mixture with DIM or ICI and E2 compared to E2 alone (*p* < 0.05 according to Dunnett’s multiple comparison test); and c: significant reduction in expression of each protein by the mixture with DIM or ICI and CYP compared to CYP alone (*p* < 0.05 according to Dunnett’s multiple comparison test).

To clarify the effects of E2 and CYP on protein expression of metastasis-related genes, such as Cathepsin D and MMP-9, a Western blot assay was conducted. In cells treated with CYP (10^−8^ M), the protein expression of Cathepsin D and MMP-9 increased by 67% (Cathepsin D) and by 79% (MMP-9), similar to E2 (10^−9^ M; 47% for Cathepsin D and 55% for MMP-9) compared to a control (Figure 6). Conversely, when cells were co-treated with ICI 182,780 (10^−8^ M) or DIM (10^−7^ M), the expression of Cathepsin D and MMP-9 was restored to the control level (Figure 6A–C). These results indicate that the metastasis of Ishikawa cancer cells may be induced by E2 or CYP through increased expression of Cathepsin D and MMP-9 protein in Ishikawa cells via the ER-dependent signaling pathway, and that DIM can suppress the metastatic potential of Ishikawa cells.

## 3. Discussion

Cancer metastasis, which is the spread of cancer cells by a cancerous tumor within the body, is the primary cause of cancer mortality [28]. In the initiation step of the metastatic process, EMT enables tumor cells to acquire migratory and invasive capabilities through the formation of motile characteristics [28,29]. In estrogen-dependent cancers, E2 was found to increase the metastatic potential by inducing EMT, migration, and invasion of cancer cells via the ER-dependent pathway [21,30,31]. In this regard, EDCs having estrogenic activity are also implicated with cancer progression and metastasis of estrogen-dependent cancers [30,32]. As typical EDCs, bisphenol A, and nonylphenol were reported to enhance the EMT process and migration of ovarian cancer cells via the ER-dependent pathway [12]. Conversely, phytoestrogens exerted anti-metastatic effects by inhibiting EMT, migration and the invasion of estrogen-responsive cancer cells, which is associated with their anti-estrogenic activity. For instance, genistein suppressed E2-induced EMT and the migration of ER-positive BG-1 ovarian cancer cells [33]. Moreover, kaempferol restrained E2-induced EMT and the metastatic behaviors of ER-positive MCF-7 breast cancer cells [10].

In the present study, we investigated the concurrent effects of CYP as an EDC and DIM as a phytoestrogen on the cell viability, migration, and invasion capacities of Ishikawa endometrial cancer cells that are estrogen responsive. A cell viability assay showed that treatment with E2 (10^−9^ M) or CYP (10^−10^–10^−6^ M) increased the level of cell viability of Ishikawa cells. In addition, E2 (10^−9^ M) and CYP (10^−8^ M) changed the cell morphology of Ishikawa cells from a cobblestone appearance, which is a typical morphology of epithelial cells, to a spindle-shaped morphology or a fibroblast-like morphology, which is typical of mesenchymal cells in the ER-dependent pathway. In the present study, ER dependency of E2 and CYP was identified by co-treatment with ICI 182,780, an ER antagonist, which counteracted the effects of E2, as well as CYP. In addition, E2 and CYP decreased the protein expression of E-cadherin, but increased the expression of N-cadherin and Snail. E-cadherin as a typical epithelial cell marker is a transmembrane protein that is responsible for the adherens junction [34]. During the progression of invasive carcinoma, E-cadherin loss is permitted for a crucial stage causing the EMT event [35]. However, N-cadherin and Snail as mesenchymal cell markers assign an invasive capacity for metastasis to the cancer cells [36,37,38]. Over-expression of N-cadherin is associated with an invasive capacity of breast tumor by increasing the interactions between tumor cells and stromal cells [39]. Based on the ability to induce the EMT process, E2 and CYP were found to promote the migration and invasion of Ishikawa cancer cells and to increase the protein expression of metastasis-related genes, such as Cathepsin D and MMP-9 [40,41], in the ER-dependent pathway. Therefore, these results indicate that CYP may induce metastatic processes of endometrial cancer cells including EMT, migration, and invasion, similar to E2. In our previous study, CYP was found to enhance cell cycle progression and cell migration in an estrogen-responsive ovarian cancer model in an ER signaling-dependent manner [24].

On the contrary, when DIM was co-treated with E2 or CYP, DIM withdrew E2 and CYP-induced cell viability, EMT, migration, and invasion of Ishikawa endometrial cancer cells, even at the low concentration of 10^−7^ M. This effect of co-treatment of DIM was similar to that of co-treatment of ICI 182,780 with E2 or CYP, indicating that DIM, as a phytoestrogen, has an anti-estrogenic activity that is associated with its anti-metastatic potential to suppress E2 or CYP-induced metastasis of estrogen-dependent endometrial cancer. A previous study reported that dietary I3C, a precursor substance of DIM, prevents the development of estrogen-enhanced cancers as a negative regulator of estrogen [42]. As anti-estrogens, I3C and DIM are known to have anti-tumorigenic properties by targeting ER-alpha (ER-α), and DIM was more effective than I3C at depressing mRNA expression of ER-α in MCF-7 breast cancer cells [43]. Additionally, in a study using thyroid cancer model, DIM was found to inhibit E2-induced proliferation and clone formation of cancer cells in a similar fashion to ICI 182,780 and act as an anti-estrogen by possibly targeting E2-ER signaling pathways [44]. Although the more detailed mechanism for anti-estrogenic properties of DIM in connection with its anti-EMT and anti-metastatic potential in endometrial cancer was not identified in the present study, DIM was found to suppress endometrial cancer metastasis by abrocating the effects of E2 and CYP in a similar way to anti-estrogen.

**Figure 7:**
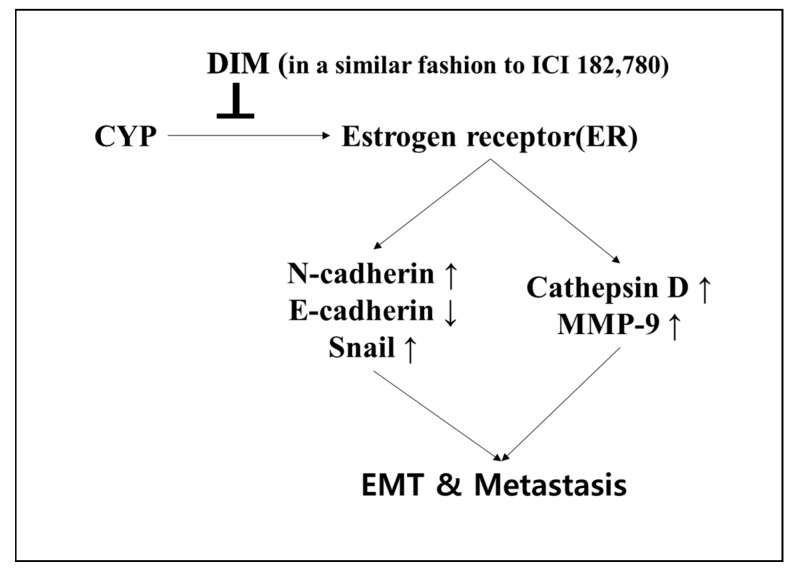
Suppressive behaviors of DIM on E2- or CYP-induced EMT and metastasis of Ishikawa cells. CYP was presented by acting as xenoestrogens via acceleration of the proliferation of estrogen-responsive Ishikawa endometrial cancer cells. CYP also enhanced EMT, migration, and invasion of Ishikawa cells by regulating EMT-related genes, such as *E-cadherin*, *N-cadherin*, and *Snail*, as well as metastasis-related genes, including *Cathepsin D* and *MMP-9* in an ER-dependent manner, as did E2. Conversely, DIM was found to significantly suppress E2 and CYP-induced proliferation, EMT, migration, and invasion of Ishikawa cancer cells, implying that while CYP has the capacity to enhance the metastatic potential of estrogen-responsive endometrial cancer, DIM has an anti-estrogenic chemopreventive effect that withdraws the cancer-enhancing effect of E2 and CYP (⊥; decrease or inhibit, arrows; increase or promote).

In summary, as shown in Figure 7, CYP was shown to work as a xenoestrogen by stimulating an increase in cell viability of Ishikawa cells. Moreover, CYP promoted the ability of metastasis of Ishikawa cells by causing the EMT process, cell migration, and invasion through the regulation of E-cadherin, N-cadherin, and Snail as EMT-related markers and Cathepsin D, as well as MMP-9, as metastasis-related markers through the pathway of the ER-dependent signaling. Therefore, the present study indicated that CYP is a risk factor for endometrial cancer progression through activating ER signaling. Conversely, DIM was shown to act as an anti-cancer agent by mimicking the function of ICI 182,780 as an ER-antagonist by reducing the cancer progression effect of endogenous estrogen and exogenous EDCs. However, more studies are needed to elucidate the molecular mechanisms underlying the two conflicting effects of CYP and DIM revealed in endometrial cancer progression.

## 4. Materials and Methods

### 4.1. Reagents and Chemicals

17β-estradiol (E2), CYP, and DIM were purchased from Sigma-Aldrich Corp. (St. Louis, MO, USA), while fulvestrant (ICI 182,780) was purchased from Tocris Bioscience (Avon, Bristol, UK). All chemicals were dissolved in 100% dimethyl sulfoxide (DMSO; Junsei Chemical Co., Tokyo, Japan) and stored at room temperature.

### 4.2. Cell Culture and Media

The Ishikawa cell line was obtained from E.B. Jeung (College of Veterinary Medicine, Chungbuk National University, Cheongju, Chungbuk, Korea). Ishikawa cells were cultivated in Dulbecco’s modified Eagle’s medium (DMEM; HyClone Laboratories Inc., Logan, UT, USA) replenished with 10% heat-inactivated fetal bovine serum (FBS; RMBIO, Missoula, MT, USA), 2% penicillin, streptomycin (Capricorn Scientific, Ebsdorfergrund, Germany), and 1% HEPES (Thermofisher Scientific, Waltham, MA, USA) at 37 °C in a humidified atmosphere of 95% air-5% CO_2_. To exclude estrogenic components from DMEM and FBS, phenol red-free DMEM with 5% charcoal-dextran processed FBS (CD-FBS) was utilized to cultivate Ishikawa cells and to estimate the estrogenicity of EDCs. The CD-FBS was made in the laboratory as follows. Approximately 40 mL of distilled water (DW) was added to 2.2 g of charcoal in a 50 mL conical tube (SPL Life Science, Seoul, Korea), mixed strongly, and centrifuged at 300 rpm for 5 min. After dropping DW into the tube, 40 mL of the new DW was added. This process was repeated 20 times, after which the DW was discarded and 0.22 g of dextran was added to the tube. Next, 40 mL of DW was added and centrifuged for 5 min at 300 rpm with strong inverting. After discarding all DW in the tube, 40 mL of new DW was added. This process was repeated 20 times. Once clear, all DW was discarded. Next, 42 mL of inactivated FBS was added and the samples were inverted strongly. Samples were then filtered twice with a 0.22 µm bottle filter (Millipore, Billerica, MA, USA), after which they were stored at −20 °C until use. Ishikawa cells were cultured in phenol red-free DMEM with 5% CD-FBS for conducting the diverse assays tested in the present study. The cells were detached with 0.05% Trypsin-EDTA (Life Technologies, Carlsbad, CA, USA).

### 4.3. Cell Viability Assay

A cell viability assay was conducted to estimate the influence of E2, CYP, and DIM on Ishikawa cell proliferation. Ishikawa cells were implanted in 96-well plates (SPL Life Science) at a density of 3 × 10^3^ cells per well at 37 °C in a humidified atmosphere supplemented with 5% CO_2_. For 48 h, the cells were cultivated in phenol red-free DMEM with 5% CD-FBS. Samples were then treated with diverse concentrations of E2, CYP, or DIM (E2: 10^−9^ M, CYP: 10^−11^–10^−5^ M, or DIM: 10^−8^–10^−6^ M) in phenol red-free DMEM with 5% CD-FBS for six days. The media were switched, replaced with identical new media every two days during this period. When adding chemicals to the media, 0.1% DMSO was utilized as a vehicle. Cell viability was detected by the addition of EZ-cytox (DOGEN, Cheongju, Chungbuk, Korea). Briefly, EZ-cytox solution diluted 1:10 was added to each well of a 96-well plate, after which samples were incubated for 90 min at 37 °C in a humidified atmosphere supplemented with 5% CO_2_. The optical density (OD) per well was monitored at 450 nm using an Epoch (BioTek, Winooski, VT, USA) to determine the number of viable cells.

### 4.4. Protein Extraction and Western Blot Assay

Ishikawa cells were cultivated in 100 mm dishes to a density of 1.0 × 10^6^ cells, then treated with 0.1% DMSO (control), E2 (10^−9^ M), CYP (10^−8^ M), DIM (10^−7^ M), or ICI 182,780 (10^−8^ M) or combinations of ICI 182,780 (10^−8^ M) and E2, CYP or DIM. After treatment with chemicals, the proteins from Ishikawa cells were yielded with RIPA lysis buffer (pH 8.0, 50 mM Tris-HCl; 0.1% SDS, 0.5% deoxycholic acid, 1% NP-40, and 150 mM NaCl). Total protein concentrations were measured through utilization of bicinchoninic acid (BCA; Sigma-Aldrich Corp.). Briefly, proteins on 10% SDS-PAGE gel were transferred to a polyvinylidene fluoride (PVDF) membrane (BioRad Laboratories, Hercules, CA, USA). The membrane was then cultivated with primary antibody (Table 1) at 4 °C overnight. Primary antibody binding was identified using horseradish peroxidase (HRP) conjugated with secondary antibody (anti-rabbit lgG (H + L) or goat anti-mouse lgG (H + L); 1:5000 dilution, BioRad Laboratories). Aimed proteins were detected using WSE-7120 EzWestLumi plus (ATTO, Motoasakusa, Taito-ku, Tokyo, Japan). Individual proteins were quantified by scanning the band density on a transfer membrane using Lumino Graph II (ATTO).

### 4.5. Effects of E2, CYP, or DIM on Ishikawa Cells Morphology

Ishikawa cells were seeded into 6-well plates, then treated with E2 (10^−9^ M), CYP (10^−8^ M), or combinations of ICI 182,780 (10^−8^ M) or DIM (10^−7^ M) and E2 or CYP for 24 h. Before and after treatment, samples were viewed under a microscope (Olympus IX-73 Inverted Microscopy, Olympus, Tokyo, Japan) at 400× magnification.

### 4.6. Scratch-Wound Healing Assay

Ishikawa cells were cultivated in six-well plates at 37 °C in a humidified atmosphere supplemented with 5% CO_2_ until over 70% confluent (approximately 1.0 × 10^−6^ cells). Monolayers of Ishikawa cells implanted in wells were scratched to the same width and length using a 1 mL micropipette tip, then treated with media including 5% CD-FBS with 0.1% DMSO as a control, E2 (10^−9^ M), CYP (10^−8^ M), DIM (10^−7^ M), or ICI 182,780 (10^−8^ M), or a combination of E2 (10^−9^ M), CYP (10^−8^ M), DIM (10^−7^ M), or ICI 182,780 (10^−8^ M), respectively, then incubated for 48 h. In each treatment group, the images were viewed under 40× magnification using an Olympus IX-73 Inverted Microscope (Olympus).

### 4.7. Data Analysis

All experiments were repeated at least three times, and the data were analyzed using the Graph-pad Prism software (San Diego, CA, USA). Data were presented as the means ± SD and statistically analyzed as one-way analysis of variance (ANOVA) followed by Dunnett’s multiple comparison test. *p*-Values < 0.05 were regarded as statistically significant.

## Figures and Tables

**Table 1:** Antibodies utilized in this study.

Protein	Company	Cat No.	Description	Dilution Ratio
E-cadherin	Abcam	Ab15148	Rabbit pAb	1:500
Occludin	Santa Cruz	Sc-5562	Rabbit pAb	1:1000
N-cadherin	Abcom	Ab98952	Mouse mAb	1:2000
Snail	Cell signaling	3895S	Mouse mAb	1:1000
Cathepsin D	Abcam	Ab75852	Rabbit mAb	1:2000
MMP-9	Abcam	Ab76003	Rabbit mAb	1:1000
GAPDH	Abcam	Ab8245	Mouse mAb	1:2000
